# Improvement of the activity of the anti-HIV-1 integrase aptamer T30175 by introducing a modified thymidine into the loops

**DOI:** 10.1038/s41598-018-25720-1

**Published:** 2018-05-10

**Authors:** Antonella Virgilio, Teresa Amato, Luigi Petraccone, Francesca Esposito, Nicole Grandi, Enzo Tramontano, Raquel Romero, Shozeb Haider, Isabel Gomez-Monterrey, Ettore Novellino, Luciano Mayol, Veronica Esposito, Aldo Galeone

**Affiliations:** 1 Department of Pharmacy, University of Naples Federico II, Via D. Montesano 49, I-80131 Naples, Italy; 2 Department of Chemical Sciences, University of Naples Federico II, Via Cintia, I-80126 Naples, Italy; 3 Department of Life and Environmental Sciences, University of Cagliari, Cittadella Universitaria di Monserrato, 09045 Monserrato, Italy; 4 University College London-School of Pharmacy, 29-39 Brunswick Square, London, WC1N1AX UK

## Abstract

In this paper, we report our investigations on analogues of the anti-human immunodeficiency virus type 1 (HIV-1) integrase (IN) aptamer T30175 in which the individual thymidines forming the loops were replaced by 5-hydroxymethyl-2′-deoxyuridine residues (**H**). Circular dichroism, nuclear magnetic resonance and gel electrophoresis investigations clearly indicated that all the modified aptamers preserve the ability to form the original 5′-5′ end-stacked head-to-head dimeric G-quadruplex structure, in which each G-quadruplex adopts a parallel arrangement and is characterized by three G-tetrads, three propeller loops and one bulge-loop. All the modified aptamers were tested in an IN inhibition LEDGF-independent assay. While the modified aptamers **INTB**-**H13** and **INTB**-**H17** showed IC_50_ values comparable with that of the parent aptamer (**INTB**-**nat**), analogues **INTB**-**H2**, **INTB**-**H5** and, to a lesser extent, **INTB**-**H9** showed a higher ability to inhibit the HIV IN than the unmodified aptamer. Molecular modelling studies evaluating the aptamer/HIV IN interaction highlighted the ability of the modified thymidines to establish several contacts with the target protein. All the data point to the importance of loops in the aptamer/target interaction and suggest that the site-specific replacement of loop residues with commercially available analogues can be considered a straightforward strategy to improve the biological activities of several G-quadruplex aptamers.

## Introduction

Aptamers can be defined as relatively small nucleic acid sequences able to bind with a high affinity and specificity to particular target molecules such as small molecules, peptides, proteins etc.^1–3^. Although they are generally selected by a number of combinatorial techniques, overall called SELEX, the word “aptamer” has been extended also to target-recognizing nucleic acid fragments that were found by different methods^4^. One of the crucial properties of such ligands is their thermodynamic stability. Aptamers fold in specific secondary structures, which are usually stabilized by Watson-Crick and/or non-canonical base couplings. Therefore, it is not particularly surprising that several aptamers adopt G-quadruplex structures, belonging to one of the most stable nucleic acid secondary conformations^5,6^. The folding in G-quadruplex conformations requires G-rich sequences that are able to form square planar arrangements of four guanosines (known as G-tetrads) linked through eight H-bonds overall. The stacking of two or more G-tetrads and the presence of a metal cation in between them, further contribute to stabilize the structure. Most G-quadruplex aptamers, whose structure has been ascertained or hypothesized, are characterized by the presence of one- or two-residue loops connecting the G-runs and protruding outwardly. This category includes the anti-HIV-1 IN aptamers T30923^7^ (also endowed with an affinity to the interleukin-6 receptor)^8^, T30175^9^ (and their versions containing phosphorothioate linkages, namely T30695 and T30177, respectively) and 93del^10,11^, the thrombin-targeting anticoagulant aptamers TBA^12^ and NU172^13^, the anti-STAT3 aptamer T40214^7,14^ and the nucleolin-targeting antiproliferative and anti-HIV-1 aptamer AS1411^15,16^. Taking into account their type of folding, it is quite reasonable to assume that most of the structural stability in these G-quadruplex aptamers relies on the compact core formed by the stacked G-tetrads, while the external more accessible loop residues are mostly responsible for the interaction with the target protein.

The HIV-1 IN viral-coded protein is a dimer-of-dimers^17^. The core and C-terminal DNA binding domain, the last one resembling an SH3 domain, exhibit non-specific but strong DNA binding activity^18^. HIV-1 IN allows the integration of the HIV-1 genome into the host cell chromosome, and represents a well established viral molecular target that has been already explored for the development of small molecules binding either to its catalytic site^19^ or to allosteric binding sites^18,20^. Despite the fact that IN inhibitors are already available for therapy, the identification of new drugs with an innovative mode of action and possibly able to overcome the selection of drug resistant strains, is still an emergency^21–25^. Among these, aptamers are a very promising class of IN inhibitors. Furthermore, the positively charged cavity formed at the dimer-of-dimers interface in the crystal structure of the HIV-1 IN (PDB code 1K6Y) has also been suggested as a suitable site for DNA and 93del aptamer binding^10,17,26^.

In general, the initial natural sequence of an aptamer is later subjected to several chemical modifications aimed at improving its thermal stability, enhancing the affinity and specificity to the target, increasing the resistance in biological environments and obtaining useful data concerning the interaction with the target^27^. From this point of view, the TBA (Thrombin Binding Aptamer) represents the most obvious example, since it has undergone a plethora of chemical modifications concerning, in particular, its loops which have been proven to be responsible for the interaction with the thrombin^12^.

**Figure 1:**
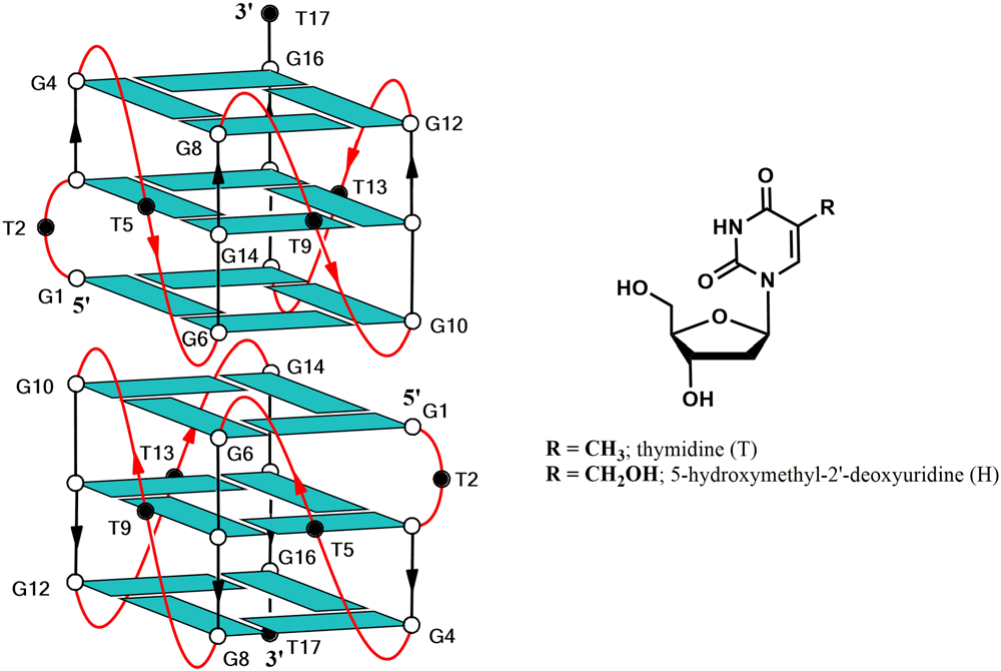
Schematic representation of the dimeric G-quadruplex structure adopted by aptamer T30175 and chemical structure of thymidine (T) and 5-hydroxymethyl-2′-deoxyuridine (H), introduced in positions 2, 5, 9, 13 and 17. All the guanosines adopt *anti* glycosidic conformations (in light blue). The thymidines are represented as black circles.

In contrast to the large number of investigations regarding TBA analogues and derivatives, only a few similar studies have concerned other aptamers. For example, in the cases of aptamers T30923, T30175 and 93del only a few research studies concerning site-specific modifications are known^9,10^, although the importance of the loops in the interaction with their common target, namely the HIV-1 IN, has been clearly suggested^26,28^. The structure of aptamer T30923 [(GGGT)_4_]^7,29^ and that of its strictly correlated analogue T30175 [GTGGT(GGGT)_3_]^9^ have been investigated by CD, NMR, gel electrophoresis and molecular dynamics simulations. They both form a head-to-head dimer of two identical 5′-5′ end-stacked parallel G-quadruplexes, each characterized by three G-tetrads and three propeller loops consisting of only one thymidine. In particular, compared to T30923, each G-quadruplex forming the dimer T30175 is characterized by a further bulge-loop formed by the extra thymidine in the second position of the sequence (Fig. 1). Therefore, considering its dimeric nature, the anti-HIV IN aptamer T30175 is overall characterized by eight one-thymidine loops, which can be regarded as potential sites of interaction with the target protein. These considerations make the loop thymidine residues of T30175 ideal candidates for structure-activity relationship investigations based on site-specific replacements and aimed at improving the biological activity and obtaining data about the ligand-target interaction. A further advantage in these investigations is the commercial availability of several thymidine analogues, which facilitates the exploration of the chemical space. In this frame we have chosen the thymidine analogue 5-hydroxymethyl-2′-deoxyuridine (**H**) taking into account the following features: 1) the presence of a hydroxymethyl group is a minor change, compared with its canonical counterpart, thus affecting minimally the structure of the base; 2) it changes the lipophilic nature of the methyl group in 5 to the hydrophilic one of the hydroxymethyl group, which is potentially able to form polar interactions and/or an H-bond with the target, as both donor and acceptor and 3) it has been successfully introduced in TBA, thus improving its biological activity^30^.

In this paper, we report CD, NMR, PAGE, molecular modelling and biological evaluation studies of five T30175 analogues prepared by the site-specific replacement, one by one, of each thymidine in the sequence with a 5-hydroxymethyl-2′-deoxyuridine residue, in comparison with their natural counterpart (**INTB**-**nat**) (Table 1 and Fig. 1). The collected data indicate that, although the presence of the modified nucleoside does not affect the structural features characteristic of T30175, a remarkable improvement of the biological activity was observed, depending on the **H** residue sequence position.

**Table 1:** Name, sequence, melting temperature and binding energy to HIV-1 IN of the ODNs investigated.

Name	Sequence*	T_m_ (°C) (±1)	Binding energy (Kcal/mol)
**INTB**-**nat** (T30175)	5′-GTGGTGGGTGGGTGGGT-3′	61	−110.0
**INTB**-**H2**	5′-GHGGTGGGTGGGTGGGT-3′	63	−139.1
**INTB**-**H5**	5′-GTGGHGGGTGGGTGGGT-3′	62	−137.2
**INTB**-**H9**	5′-GTGGTGGGHGGGTGGGT-3′	61	−133.4
**INTB**-**H13**	5′-GTGGTGGGTGGGHGGGT-3′	63	−132.2
**INTB**-**H17**	5′-GTGGTGGGTGGGTGGGH-3′	59	−132.9
**TT**-**INTB**-**nat**	5′-TTGTGGTGGGTGGGTGGGT-3′	74	N.T.

*H = 5-hydroxymethyl-2′-deoxyuridine; see main text for details. N.T. = not tested.

## Results

### Structural features and stability of the ODNs investigated

**Figure 2:**
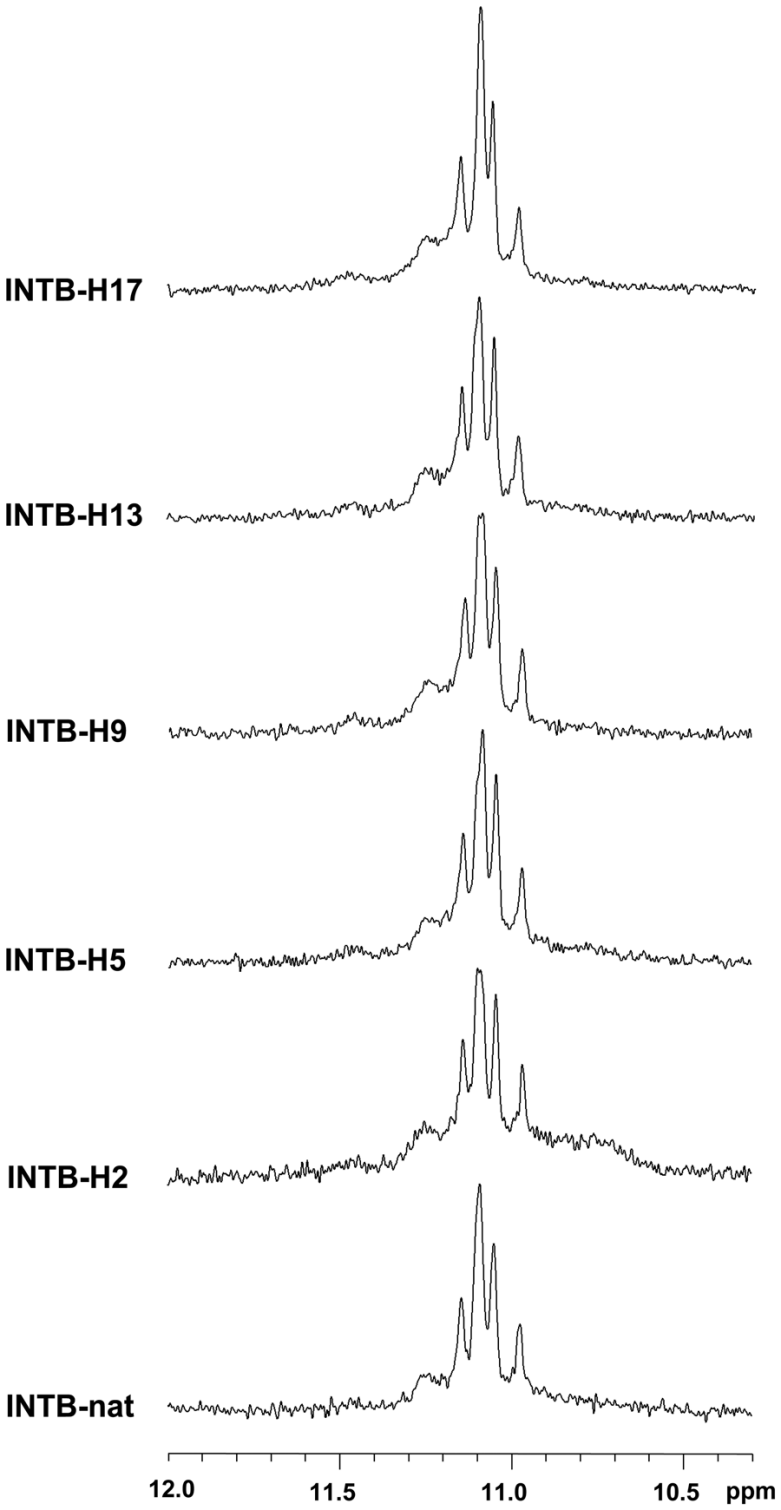
Imino proton regions of the ^1^H-NMR spectra (500 MHz) of the ODNs investigated. See main text for details.

An important issue of this investigation is to verify if the modified ODNs are able to fold in a parallel G-quadruplex structure and form the dimer characteristic of the natural parent sequence. In order to address this point, the modified T30175 analogues were analyzed by ^1^H-NMR and compared with their unmodified counterpart (Fig. 2). A straightforward comparison of the imino proton regions diagnostic of the presence of G-quadruplex structures (10.5–12.0 ppm) strongly suggests that the G-quadruplex structures adopted by the modified ODNs containing an **H** residue strictly resemble that of their natural counterpart GTGGT(GGGT)_3_ (T30175).

**Figure 3:**
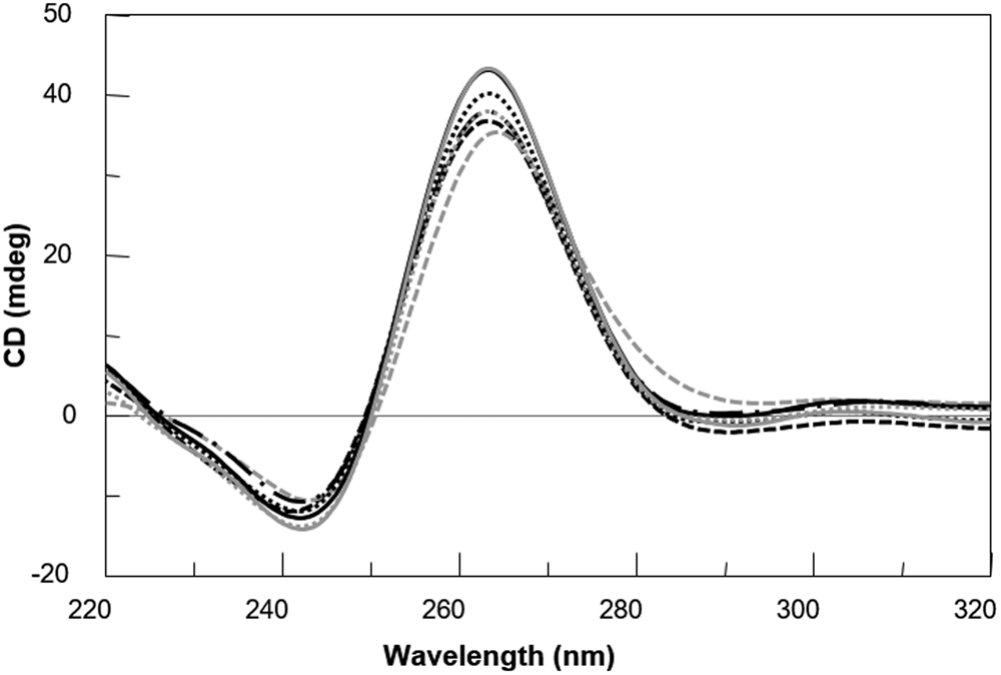
CD spectra of the ODNs investigated. Conditions: 20 °C in potassium phosphate buffer (1 mM KH_2_PO_4_/K_2_HPO_4_, 3 mM KCl, pH 7.0) at 35 µM ODN strand concentration. **INTB**-**nat** (black dashed line), **TT**-**INTB**-**nat** (grey dashed line), **INTB**-**H2** (black dashed dotted line), **INTB**-**H5** (black solid line), **INTB**-**H9** (black dotted line), **INTB**-**H13** (grey dotted line) and **INT**-**BH17** (grey solid line).

**Figure 4:**
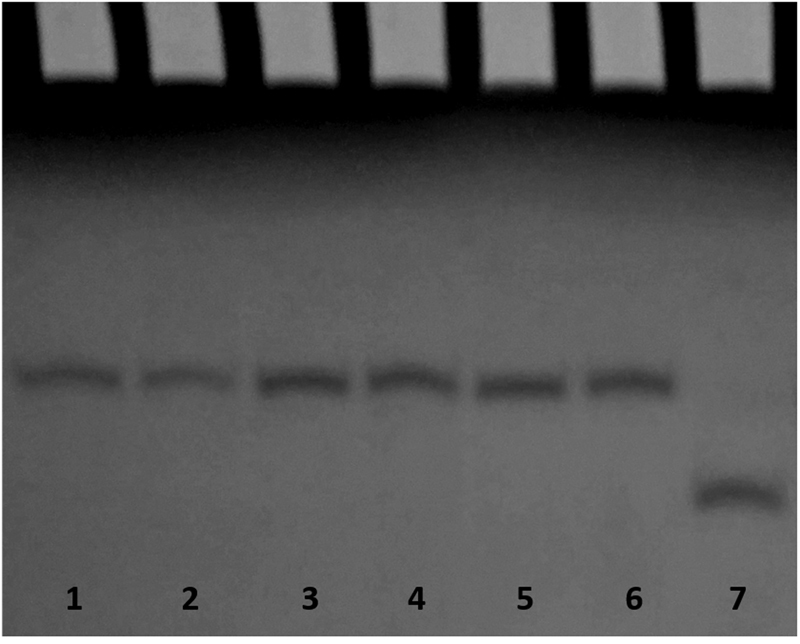
PAGE analysis of the natural and modified ODN sequences investigated. Lane 1: **INTB**-**nat**; lane 2: **INTB**-**H2**; lane 3: **INTB**-**H5**; lane 4: **INTB**-**H9**; lane 5: **INTB**-**H13**; lane 6: **INTB**-**H17**; lane 7: **TT**-**INTB**-**nat**.

With the aim of substantiating the ^1^H-NMR data, also CD spectra of the modified T30175 aptamers were acquired and compared with their natural counterpart and with the ODN TTGTGGT(GGGT)_3_ (**TT**-**INTB**-**nat**) corresponding to the sequence of T30175, extended with two extra thymidines at the 5′-end which, according to other authors^9^, prevent the formation of the 5′-5′ head-to-head dimer (Fig. 3). The unmodified aptamer T30175 shows a minor negative band at 242 nm and a major positive band at 263 nm, being characteristic of parallel G-quadruplex structures in which all guanosines adopt *anti* glycosidic conformations. Apart from negligible differences in intensity, the CD profiles of the modified sequences are almost superimposable on that of the natural sequence, thus strongly suggesting that they adopt parallel G-quadruplex structures closely resembling that of the parent aptamer T30175, in agreement with the NMR results. Furthermore, the melting temperatures (T_m_) evaluated through the CD heating profiles of the modified ODNs and their natural sequence (Fig. S1) clearly showed very similar values (taking into account the experimental error) (Table 1), thus indicating that the presence of an **H** residue in the sequence does not significantly affect the conformation adopted by the original sequence and its stability in the experimental conditions used (see below). Interestingly, although the CD profile of **TT**-**INTB**-**nat** preserves the main features of an all-G-*anti* parallel G-quadruplex, it shows a slight shift of the maximum to higher wavelengths. However, since this datum does not provide clear evidence of the presence of dimers for the modified sequences, we analyzed those by PAGE (Fig. 4) in comparison with the natural sequence **INTB**-**nat** (which has been proven to form a dimer) and **TT**-**INTB**-**nat** (in which the dimer formation is prevented by the extra thymidines in 5′). The PAGE results clearly indicated that **INTB**-**nat** and all ODNs containing an **H** residue show slower-migrating bands, which have been ascribed to dimeric structures, while **TT**-**INTB**-**nat** shows a faster-migrating band, thus pointing to the presence of a monomeric G-quadruplex.

To further compare the unfolding behavior of the studied sequences, a van’t Hoff analysis on the CD melting profiles was performed on the basis of a two-states unfolding mechanism. Firstly, the enthalpy changes for the unfolding process were obtained by assuming a two-states process in which the cooperative unit is the whole dimer (i.e. the dissociation is concomitant with the unfolding process). The enthalpy values provided by this model (Table S1) range between 131 kJ/mol (**INTB**-**H5**) and 175 kJ/mol (**INTB**-**H13**). These values are surprisingly small when compared with the enthalpy change (255 kJ/mol) obtained for the single quadruplex forming sequence (**TT**-**INTB**-**nat**). Furthermore, these low enthalpy values are completely unexpected on the basis of the T30175 NMR dimeric structure showing an additional monomer-monomer interaction that should result in an extra enthalpic contribution to the monomer unfolding enthalpy. However, these contradictory enthalpy values could be the result of the inadequacy of the fitting model based on the assumption that the whole dimer melts cooperatively. Prompted by these considerations, we decided to fit our data taking the single quadruplex as a cooperative unit; this corresponds to assuming that the dimer dissociates at a temperature far below the observed melting temperature and that the melting profile mainly reflects the monomer unfolding. The enthalpy changes obtained with this fitting model are much more consistent with the other data (Table S1). Indeed, the enthalpy changes for the modified sequences are close (within the experimental errors) to the value obtained for the unmodified sequence, as expected on the basis of the similarity of their structure (as proven by NMR and CD). Further, the enthalpy change values are comparable with the value obtained for the **TT**-**INTB**-**nat** sequence and close to the value expected for a monomeric three-G-tetrad containing G-quadruplex^31,32^. All together, these data are consistent with the hypothesis that the chemical modification does not change drastically the T30175 structure or its unfolding mechanism.

### Evaluation of the anti-HIV IN activity

A number of HIV-1 IN inhibitors have been identified so far either able to interfere with the HIV-1 IN catalytic activity or able to act as allosteric inhibitors. These inhibitors include small molecules^24,25,33^, peptides^34^ and natural compounds^21,22,35^. In addition, G-rich oligonucleotides have been shown to inhibit HIV1-IN activity at nanomolar concentrations^10^. Moreover, analogues obtained by modifying individual loop residues from the anti-HIV-1 IN aptamer 93del, were able to affect the HIV-1 IN reactions^10^. Hence, the modified T30175 analogues containing **H** residues were tested *in vitro* for their ability to inhibit HIV-1 IN activities, using the strand transfer inhibitor Raltegravir as a positive control. The results showed that all the modified analogues potently inhibited the HIV-1 IN activities (Table 2). In particular, **INTB**-**H2** and **INTB**-**H5** inhibited the HIV1-IN LEDGF independent activities with IC_50_ values of 0.145 and 0.150 µM, respectively, while **INTB**-**H9** inhibited the HIV-1 IN functions with an IC_50_ value of 0.178 µM. Differently, for **INTB**-**H13** and **INTB**-**H17**, the observed IC_50_ values were similar to the one reported for their natural counterpart (Table 2). In accordance with the data present in literature, the modified analogues were able to inhibit the HIV-1 IN activities at submicromolar concentrations^10^.

**Table 2:** ODN inhibition of the HIV-1 IN LEDGF/p75-independent activity.

Compound	^a^IC_50_ IN LEDGF-independent integration (µM)
**INTB**-**H2**	0.145 ± 0.005
**INTB**-**H5**	0.150 ± 0.030
**INTB**-**H9**	0.178 ± 0.002
**INTB**-**H13**	0.225 ± 0.005
**INTB**-**H17**	0.225 ± 0.045
**INTB**-**nat**	0.270 ± 0.020
**Raltegravir**	0.058 ± 0.010

^a^Compound concentration required to inhibit the HIV-1 IN catalytic activities, in the absence of LEDGF, by 50%. Raltegravir has been used as a reference. See main text for details.

### Docking studies of the interaction aptamer/HIV-1 integrase

In general, the docking studies have shown that the residues **H** in the different modified aptamers are able to establish several contacts with both chains A and C of the target HIV-1 IN. The unmodified parent aptamer **T30175** presented both the highest IC_50_ (0.27 ± 0.02) µM and the least favourable complex binding energy (−110 Kcal/mol). The interactions observed by the aptamers are as follows:

#### INTB-H2

**Figure 5:**
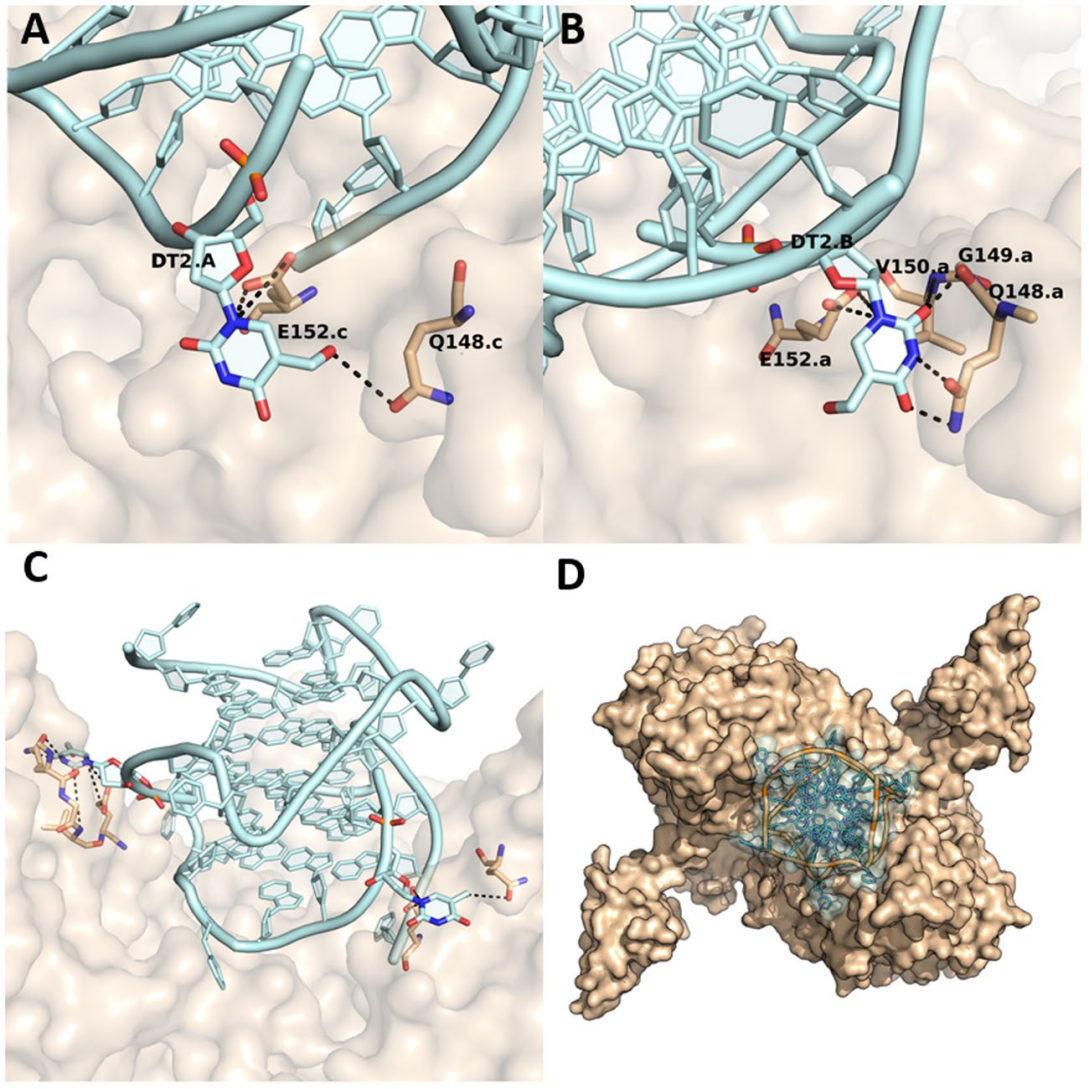
(**A**) Details of the interactions formed by the **H** residue at position 2 in chain A and (**B**) chain B of the aptamer **INTB**-**H2**. (**C**) Side view and (**D**) top view of the protein-aptamer complex. HIV1-IN has been coloured beige and represented as a surface, the aptamer **INTB**-**H2** has been represented as a cartoon and coloured in light cyan.

The **H** residue in position two in chain A of the aptamer **INTB**-**H2** makes hydrogen bonds with residues Q148 and E152 of chain C of HIV-1 IN. The **H** residue in chain B of the modified aptamer creates a hydrogen bond net with side-chains of the residues Q148, E152 and side-chains of the residues V150 and G149 of chain A of HIV-1 IN (Fig. 5).

#### INTB-H5

The **H** residue in position 5 in chain A of the aptamer **INTB**-**H5** does not make any interaction with the HIV-1 IN. However, the **H** residue in chain B of the DNA fragment establishes a hydrogen bond net with its surrender, making interactions with the side-chains of the residues T112, H114, K136 and E138 of the chain C of HIV-1 IN (Fig. S2).

#### INTB-H9

The **H** residue in the aptamer **INTB**-**H9** in chain A makes a hydrogen bond with residue Q44 of chain C of HIV-1 IN. The **H** residue in chain B at position 9 makes hydrogen bond interactions with the backbone atoms of residues S147 and Q146 of chain C of the enzyme (Fig. S3).

#### INTB-H13

The **H** residue in chain A of the aptamer **INTB**-**H13** creates a hydrogen bond network with residues Q62 and H114 of chain A of the HIV-1 IN. Modified residue **H** in the chain B of the aptamer makes a hydrogen bond with residue N144 of the HIV-1 IN (Fig. S4).

#### INTB-H17

The **H** residue at position 17 of chain A of the **INTB**-**H17** aptamer does not interact with HIV-1 IN. However, the modified nucleotide **H** in chain B makes multiple hydrogen bonds with residues Q53, V54 (backbone) and R144 of chain A of HIV-1 IN (Fig. S5).

#### INTB-nat

In the original T30175 aptamer (**INTB**-**nat**), where there are no modifications to the bases, the aptamer sits in the cavity between the HIV-1 IN subunits, where the O5′ end of chain A of the aptamer makes interactions with K370 of the HIV-1 IN (Fig. S6).

The final binding energies of the modified aptamers were: −110 Kcal/mol (**INTB**-**Nat**); −132.2 Kcal/mol (**INTB**-**H13**); −132.9 Kcal/mol (**INTB**-**H17**); −133.4 Kcal/mol (**INTB**-**H9**); −137.2 Kcal/mol (**INTB**-**H5**); −139.1 Kcal/mol (**INTB**-**H2**). Consequently, the affinities of the modified aptamers to HIV-1 IN were in the following order: **INTB**-**Nat** < **INTB**-**H13** < **INTB**-**H17** < **INTB**-**H9** < **INTB**-**H5** < **INTB**-**H2**, which is in good agreement with the biological activity data (Table 1).

## Discussion

Post-SELEX modifications of aptamers aimed at improving the interaction with the target, increasing the thermal stability or enhancing the resistance in biological environments, are essential for the development of these molecules as potential therapeutic agents and/or diagnostic tools. However, detailed information concerning the aptamer-target interaction, which is indispensable to avoid detrimental modifications, is rarely available and, in general, becomes accessible only a long time after the discovery of a given aptamer. Nevertheless, from this point of view, G-quadruplex aptamers represent an exception. In fact, several aptamers are known or have been suggested to adopt G-quadruplex structures in which stacked G-tetrads account principally for their high thermal stability while loops connecting G-runs, being protruded outwardly, establish interactions with the target. In these cases, the identification of the aptameric regions, namely the loops, mainly responsible for the interaction with the target, allows a straightforward exploration of the chemical space through site-specific replacements designed to improve the properties of these ligands, particularly if the aptamer is characterized by loops formed by one or two residues, which are thymidines in most cases. With the aim of applying this strategy to the G-quadruplex anti-HIV-1 IN aptamer T30175, we have prepared five analogues in which thymidines in the loops and at the 3′-end have been replaced one at a time by 5-hydroxymethyl-2′-deoxyuridine residues. NMR, CD and electrophoretic data strongly suggest that all the modified aptamers preserve the ability of the parent sequence to form a dimeric structure composed of two identical G-quadruplexes, each characterized by parallel strands, three all-*anti* G-tetrads and four one-thymidine loops (one bulge and three reversed-chain loops). The results of the HIV1-IN LEDGF independent assay have indicated that the presence of the modified residue in position 2 or 5 (aptamers **INTB**-**H2** and **INTB**-**H5**, respectively) allows a noteworthy improvement of the anti-HIV-1 IN activity, particularly considering the tiny chemical modification introduced. Molecular modelling data concerning the interaction between the modified aptamers and the target protein HIV-1 IN indicate a correlation between the binding energies and biological activities. Taking into account the wide commercial availability of modified thymidines, these encouraging results here described validate the simple strategy proposed and suggest its application to other G-quadruplex aptamers with structural features similar to T30175.

## Methods

### Oligonucleotides synthesis and purification

The oligodeoxyribonucleotides (ODNs) reported in Table 1 were synthesized on a Millipore Cyclone Plus DNA synthesizer using solid phase β-cyanoethylphosphoramidite chemistry at 15 µmol scale. The modified monomer 5-hydroxymethyl-2′-deoxyuridine (**H**) was introduced in the sequences using commercially available 5′-dimethoxytrityl-5-acetoxymethyl-2′-deoxyuridine-3′-[(2-cyanoethyl)-(N,N-diisopropyl)]-phosphoramidite (Glen Research). For ODN **INTB**-**H17** a universal support was used. The oligomers were detached from the support and deprotected by treatment with concentrated aqueous ammonia at 80 °C overnight. The combined filtrates and washings were concentrated under reduced pressure, redissolved in H_2_O, analyzed and purified by high-performance liquid chromatography on a Nucleogel SAX column (Macherey-Nagel, 1000–8/46), using buffer A: 20 mM KH_2_PO_4_/K_2_HPO_4_ aqueous solution (pH 7.0) containing 20% (v/v) CH_3_CN and buffer B: 1 M KCl, 20 mM KH_2_PO_4_/K_2_HPO_4_ aqueous solution (pH 7.0) containing 20% (v/v) CH_3_CN; a linear gradient from 0 to 100% B for 45 min and flow rate 1 ml/min were used. The fractions of the oligomers were collected and successively desalted by Sep-pak cartridges (C-18). The isolated oligomers proved to be >98% pure by NMR.

### CD spectroscopy

CD samples of ODNs reported in Table 1 were prepared at an ODN concentration of 35 µM using a potassium phosphate buffer (1 mM KH_2_PO_4_/K_2_HPO_4_, 3 mM KCl, pH 7.0) and submitted to the annealing procedure (heating at 90 °C and slowly cooling at room temperature). CD spectra of all G-quadruplexes and CD melting curves were registered on a Jasco 715 CD spectrophotometer. For the CD spectra, the wavelength was varied from 220 to 320 nm at 100 nm min^−1^ scan rate, and the spectra recorded with a response of 4 s, at 1.0 nm bandwidth and normalized by subtraction of the background scan with buffer. The temperature was kept constant at 20 °C with a thermoelectrically controlled cell holder (Jasco PTC-348). CD melting curves were registered as a function of temperature (range: 20 °C–90 °C) for all G-quadruplexes at their maximum Cotton effect wavelengths. The CD data were recorded in a 0.1 cm pathlength cuvette with a scan rate of 10 °C/h. Van’t Hoff analysis^36^ was performed on the CD melting curves assuming the dimer or the monomer as cooperative unit. The enthalpy changes (ΔH_v_._H_) provide the best fit of the experimental melting data (Table S1).

### NMR spectroscopy

NMR samples were prepared at a concentration of about 2 mM, in 0.6 mL (H_2_O/D_2_O 9:1 v/v), buffer solution having 10 mM KH_2_PO_4_/K_2_HPO_4_, 70 mM KCl and 0.2 mM EDTA (pH 7.0). All the samples were heated for 5–10 min at 90 °C and slowly cooled (10–12 h) to room temperature. The solutions were equilibrated for 24–48 hours at 4 °C. The annealing process was assumed to be complete when ^1^H NMR spectra were superimposable on changing time. NMR spectra were recorded with a Varian Unity INOVA 500 MHz spectrometer. 1D proton spectra of the samples in H_2_O were recorded using pulsed-field gradient DPFGSE for H_2_O suppression^37^. ^1^H-chemical shifts were referenced relative to external sodium 2,2-dimethyl-2-silapentane-5-sulfonate (DSS).

### Gel electrophoresis

All ODNs were analyzed by non-denaturing PAGE. Samples in the CD buffer (1 mM KH_2_PO_4_/K_2_HPO_4_, 3 mM KCl, pH 7.0) were loaded on a 20% polyacrylamide gel containing Tris–Borate-EDTA (TBE) 2.5× and KCl 5 mM. The run buffer was TBE 1× containing 10 mM KCl. For all samples, a solution of glycerol/TBE 10× was added just before loading. Electrophoresis was performed at 8 V/cm at a temperature close to 10 °C. Bands were visualized by UV shadowing.

### Homogeneous Time Resolved Fluorescence (HTRF) IN assay

Full-length HIV-1 IN protein was expressed in Escherichia coli BL21 (DE3) and purified as described^33,38^. The HIV-1 IN assay allows the measurement of the inhibition of the 3′-processing and strand transfer IN reactions and was performed with the following adaptations respect to the methodology described^38^. Briefly, 50 nM of integrase were preincubated with increasing concentration of compounds for 1 h at room temperature in a reaction buffer containing 10 mM HEPES pH 7.5, 1 mM DTT, 1% Glycerol, 40 mM MgCl_2_, 0.05% Brij-35 and 0.1 mg/ml BSA. To this mixture, 100 nM DNA donor substrate (5′-ACAGGCCTAGCACGCGTCG-Biotin-3′ annealed with 5′-CGACGCGTGGTAGGCCTGT-Biotin-3′) and 50 nM DNA acceptor substrate (5′-Cy5-ATGTGGAAAATCTCTAGCAGT-3′ annealed with 5′-Cy5-TGAGCTCGAGATTTTCCACAT-3′) and incubated at 37 °C for 90 min were added. After the incubation, Europium-Streptavidine 4 nM were added at the reaction mixture and the HTRF signal was recorded using a Perkin Elmer Victor 3 plate reader using a 314 nm for excitation wavelength and 668 and 620 nm for acceptor and donor substrates emission wavelength, respectively.

### Molecular modeling

HIV-1 IN/aptamer complex: models of the complex (enzyme HIV-1 IN with aptamers) were generated employing the docking program HEX^39^. The X-ray crystal structure of the protein HIV-1 IN (PDB id 1K6Y)^17^ was used as a receptor. The structure lacks coordinates for loops 1B/D (residues 46–56) and the catalytic loop 2B/D (residues 139–149). A complete model of HIV-1 IN was built based on the protocol described in Sgobba *et al*.^26^. The models of the modified derivatives of the T30177 aptamer were generated based on the dimeric G-quadruplex, which contained bulges (PDB 2M4P)^40^. In each monomer, one thymidine residue in each bulge was modified into 5-hydroxymethyl-2′-deoxyuridine to generate the modified derivatives. In total, two modified residues are present in each dimeric aptamer.

The HEX docking algorithm employs molecular shape comparison using spherical harmonics and polar Fourier correlations. The initial steric scan was kept at N = 18 followed by a final search at N = 25. This discards orientations where a steric clash occurs during the rigid body docking procedure that employs shape (steric) and electrostatic correlation. The centre of mass of both the protein and the DNA chain were used as centroids or origins of the geometrical operations. The receptor’s range angle was set at 60° with a rotational angle of 7.5°, whereas the ligand’s range angle was kept at 180° with a rotational angle of 7.5°. The twist angle of the ligand about the intermolecular axis was kept at 360° with a rotational angle of 5.5°. HEX offers a molecular mechanics refinement based on hydrogen bonds and soft Lennard Jones (12–6) potentials which is an adaptation of Optimized Potential for Liquid Simulations (OPLS) force field^41^. Thus, OPLS energies option was selected as a post processing method. The docking calculation was performed using 40 intermolecular separations in steps of 0.8 Å. A total of 1000 solutions were obtained for each docking. The top 25 ranked clustered solutions were analyzed by visual inspection and those with optimal cavity positioning and lowest energy were selected for energy minimization.

Energy minimization of the docked complexes was performed using the MD engine AMBER 16^42^. Modified thymines were renamed and their charge was calculated using antechamber. The parametrization was finally conducted with LEaP using FF14SB forcefield^43^ with explicit solvent and counter-ions. The energy minimization was carried out in two steps; firstly, only the water and counter ions were allowed to move while the protein-DNA complex was fixed using positional restrains of 500 Kcal/mol, performed in 1000 steps (switching from steepest descent to conjugate gradient after 500 steps); secondly, energy minimization of the complete system without any restrains in 2500 steps (switching from steepest descent to conjugate gradient after 500 steps).

## Electronic supplementary material

supplementary information
