# Supplementary material for: Improvement of the activity of the anti-HIV-1 integrase aptamer T30175 by introducing a modified thymidine into the loops
Source: Sci Rep. 2018 May 10;8:7447. doi: 10.1038/s41598-018-25720-1 (PMC5945619; doi:10.1038/s41598-018-25720-1)
Supplement: Supplementary file 1 — supplementary information [file 41598_2018_25720_MOESM1_ESM.pdf]

SUPPLEMENTARY INFORMATION

IMPROVEMENT OF THE ACTIVITY OF THE ANTI-HIV-1 INTEGRASE APTAMER T30175 BY INTRODUCING A MODIFIED THYMIDINE INTO THE LOOPS

Antonella Virgilio[1], Teresa Amato[1], Luigi Petraccone[2], Francesca Esposito[3], Nicole Grandi[3], Enzo Tramontano[3], Raquel Romero[4], Shozeb Haider[4], Isabel Gomez-Monterrey[1], Ettore Novellino[1], Luciano Mayol[1], Veronica Esposito[1]* and Aldo Galeone[1]*

***corresponding authors: galeone@unina.it (AG); verespos@unina.it (VE).

[1]Department of Pharmacy, University of Naples Federico II, Via D. Montesano 49, I-80131 Naples, Italy.

[2]Department of Chemical Sciences, University of Naples Federico II, Via Cintia, I-80126 Naples, Italy.

[3]Department of Life and Environmental Sciences, University of Cagliari, Cittadella Universitaria di Monserrato, 09045 Monserrato, Italy.

[4]University College London-School of Pharmacy, 29-39 Brunswick Square, London WC1N1AX, U.K.

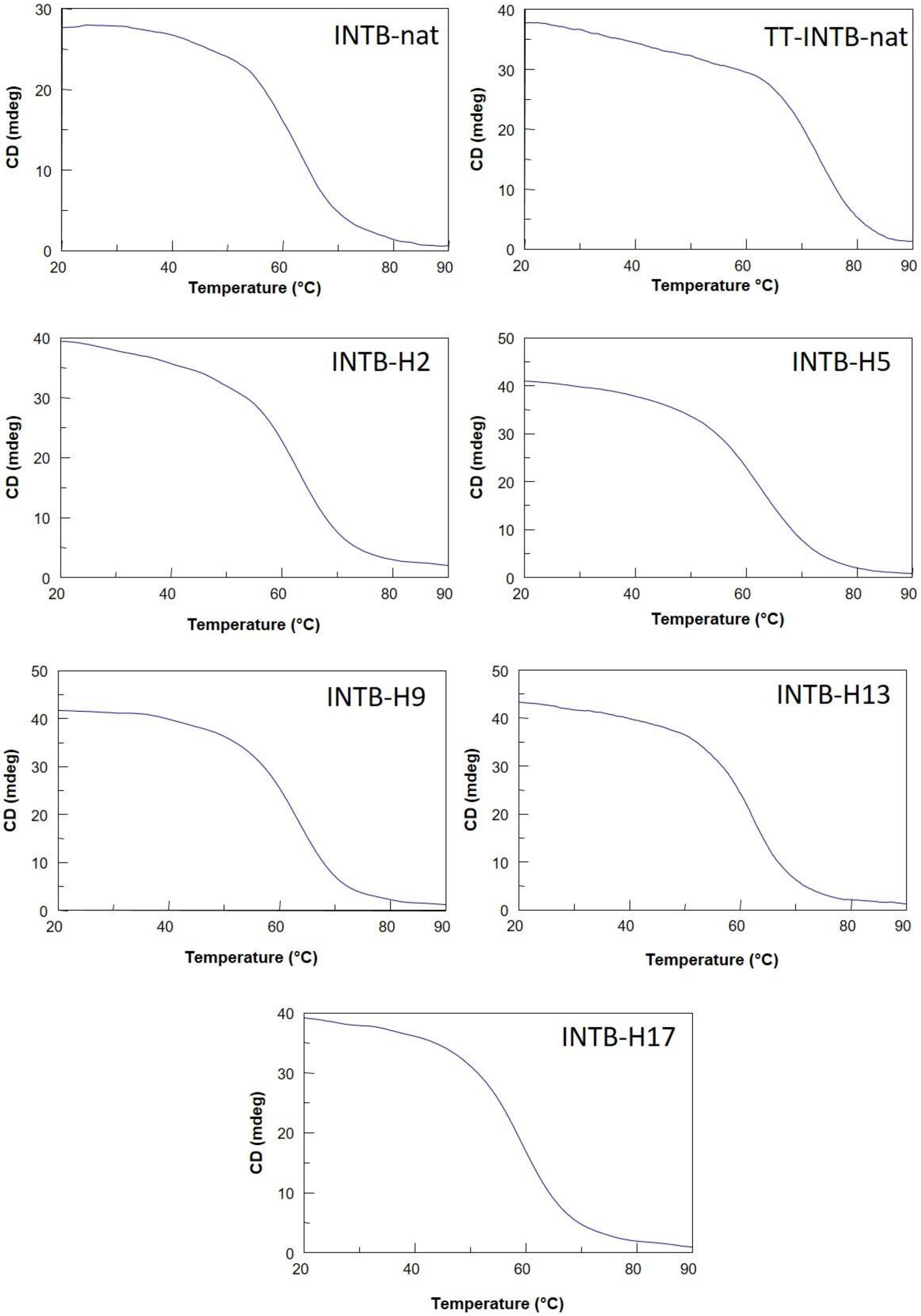

Figure S1: CD melting curves of the modified T30175 aptamers investigated and their reference ODNs **INTB-nat** and **TT-INTB-nat**.

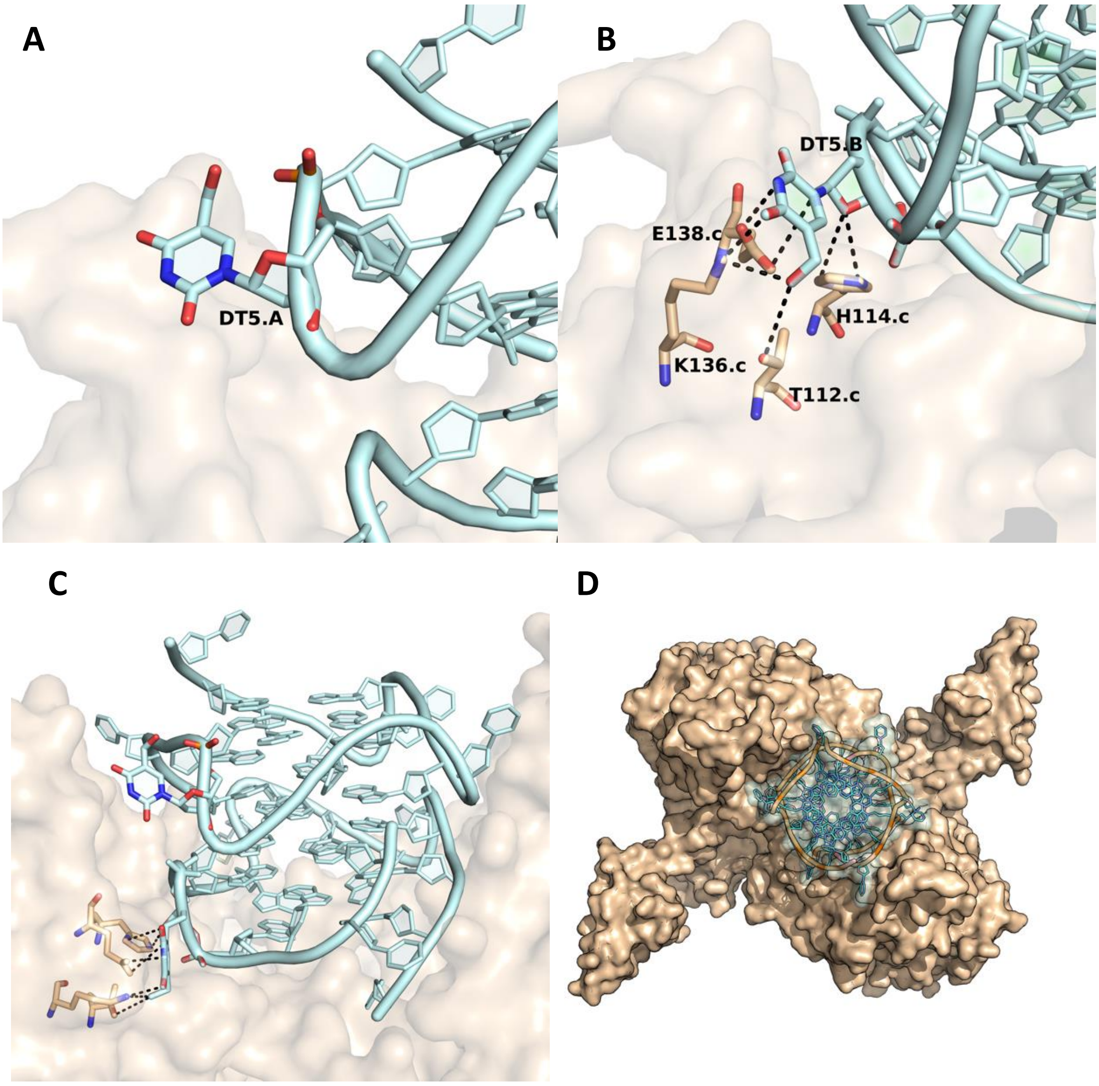

Figure S2: (A) Details of the interactions formed by the **H** residue at position 5 in chain A and (B) chain B of the aptamer **INTB-H5**. (C) Side view and (D) top view of the protein-aptamer complex. HIV-1 IN has been coloured beige and represented as a surface, the aptamer **INTB-H5** has been represented as a cartoon and coloured in light cyan.

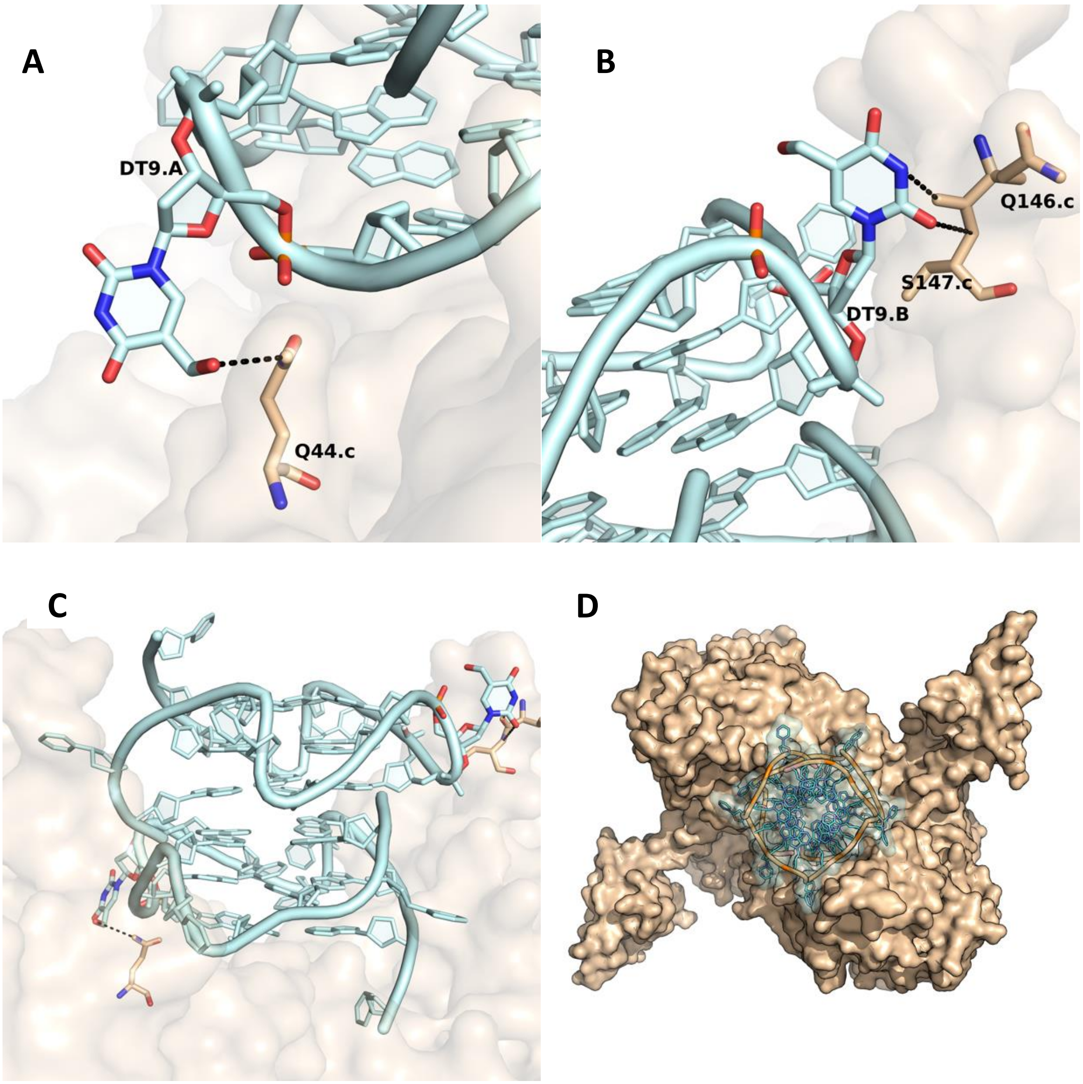

Figure S3: (A) Details of the interactions formed by the **H** residue at position 9 in chain A and (B) chain B of the aptamer **INTB-H9**. (C) Side view and (D) top view of the protein-aptamer complex. HIV-1 IN has been coloured beige and represented as a surface, the aptamer **INTB-H9** has been represented as a cartoon and coloured in light cyan.

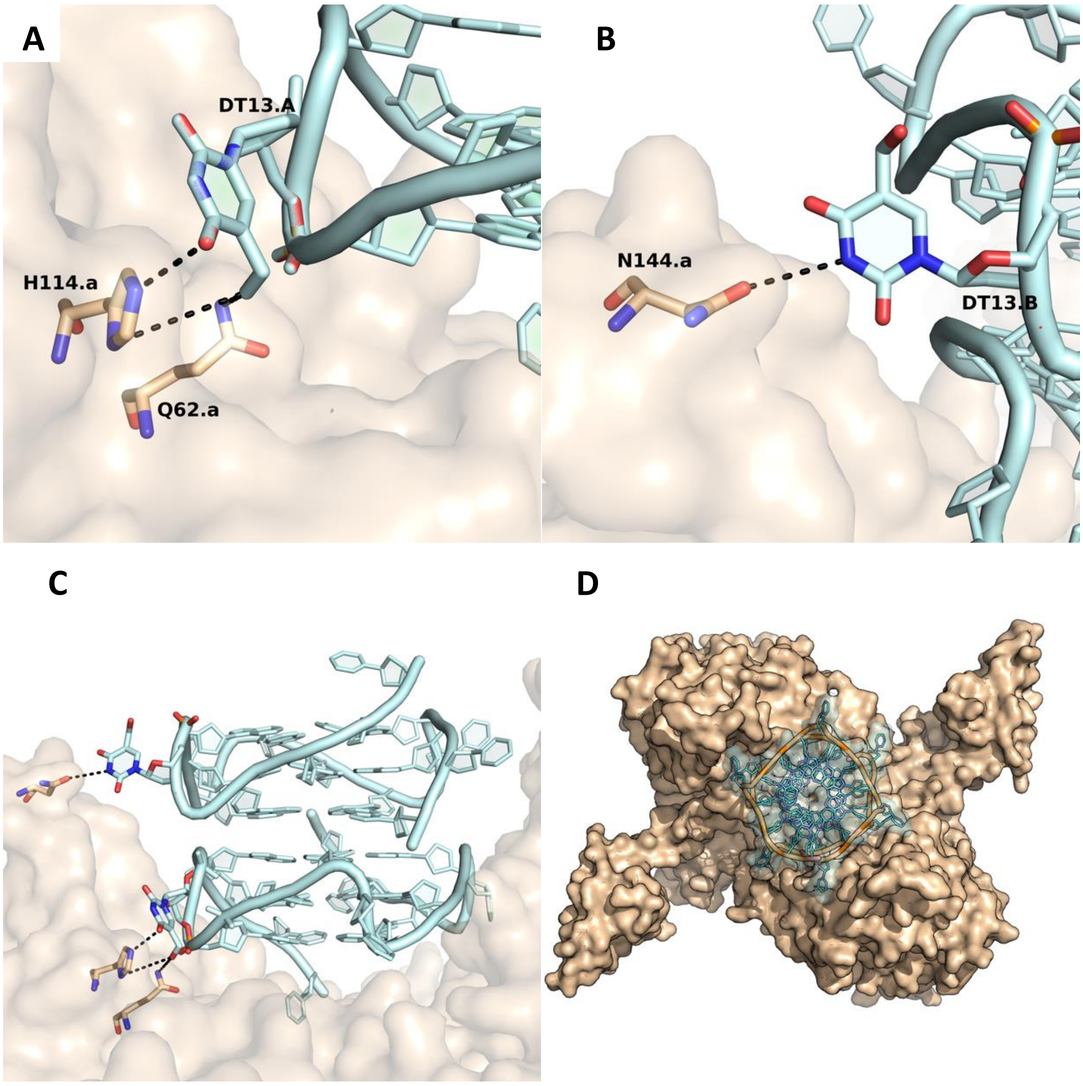

Figure S4: (A) Details of the interactions formed by the **H** residue at position 13 in chain A and (B) chain B of the aptamer **INTB-H13**. (C) Side view and (D) top view of the protein-aptamer complex. HIV-1 IN has been coloured beige and represented as a surface, the aptamer **INTB-H13** has been represented as a cartoon and coloured in light cyan.

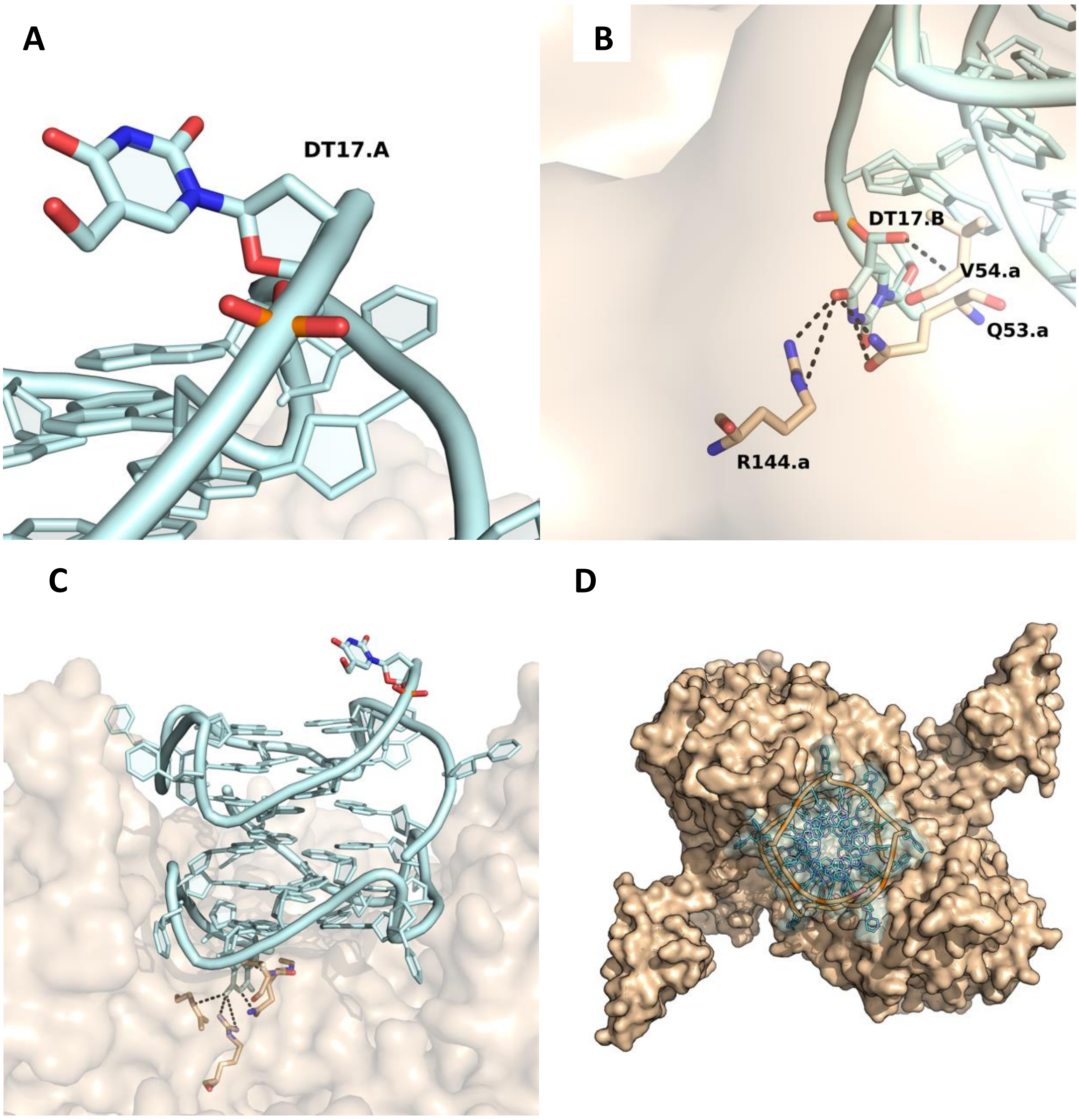

Figure S5: (A) Details of the interactions formed by the **H** residue at position 17 in chain A and (B) chain B of the aptamer **INTB-H17**. (C) Side view and (D) top view of the protein-aptamer complex. HIV-1 IN has been coloured beige and represented as a surface, the aptamer **INTB-H17** has been represented as a cartoon and coloured in light cyan.

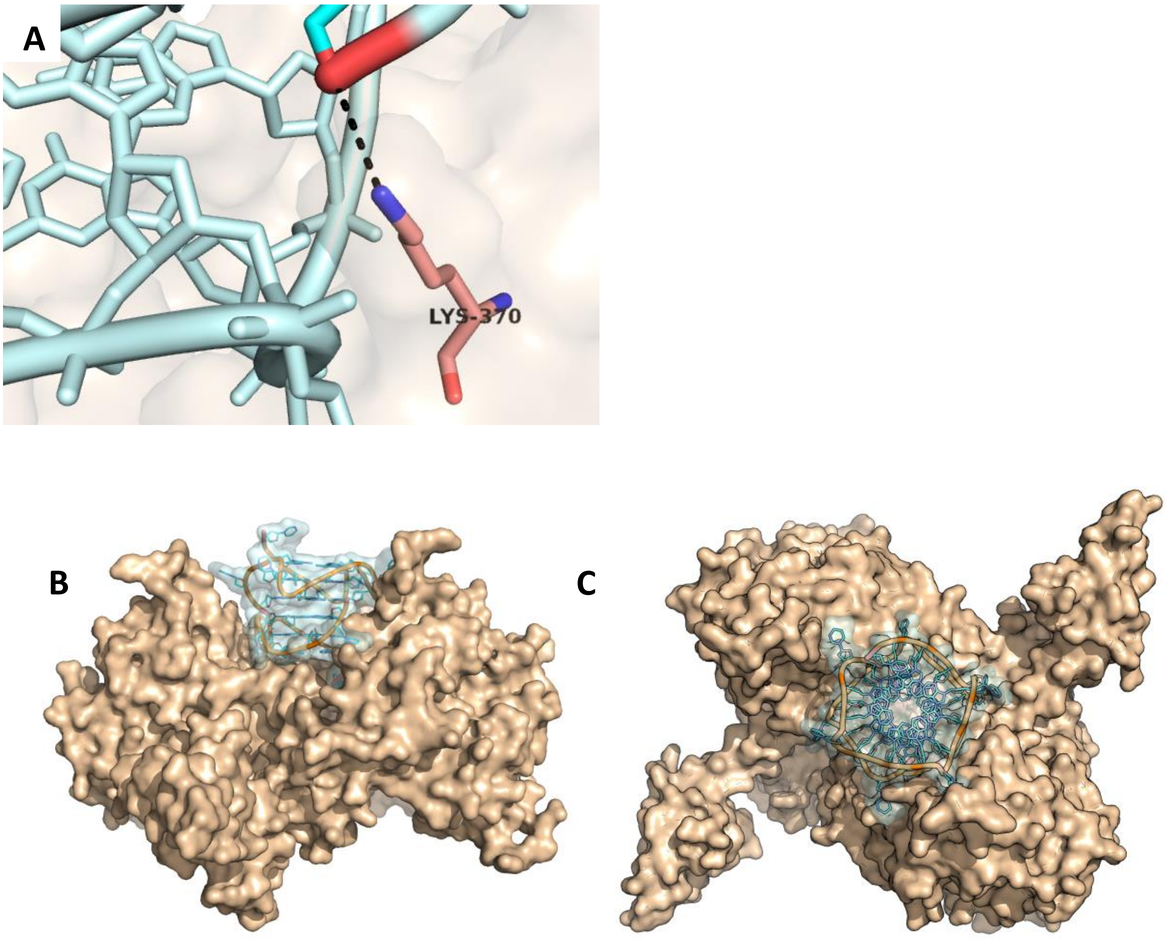

Figure S6: (A) Details of the interactions involving chain A of aptamer **INTB-nat** (A). (B) Side view and (C) top view of the protein-aptamer complex. HIV-1 IN has been coloured beige and represented as a surface, the aptamer **INTB-nat** has been represented as a cartoon and coloured in light cyan.

Sequence	$\Delta H_{v.H.}$ (kJ/mol)[a]	$\Delta H_{v.H.}$ (kJ/mol)
	Unfolding model	
	$Q_2 \longrightarrow 2S$	$Q \longrightarrow S$
INTB-nat (T30175)	154	225
INTB-H2	167	220
INTB-H5	131	200
INTB-H9	148	205
INTB-H13	175	238
INTB-H17	155	210
TT-INTB-nat	-	255

[a] Enthalpy changes are reported per mole of monomer.
Errors on ΔH_{vH} is within ±10%.

Table S1. Enthalpy changes obtained from the van't Hoff analysis of the CD melting curves by using different unfolding models.
